# Solid-phase microextraction fiber development for sampling and analysis of volatile organohalogen compounds in air

**DOI:** 10.1186/s40201-014-0123-5

**Published:** 2014-09-17

**Authors:** Seyed Ghavameddin Attari, Abdolrahman Bahrami, Farshid Ghorbani Shahna, Mahmoud Heidari

**Affiliations:** Department of Occupational Health, School of Public Health, Hamadan University of Medical Sciences, Hamadan, Iran; Department of Occupational Health, School of Health, Guilan University of Medical Sciences, Rasht, Iran

**Keywords:** Solid phase microextraction, Single walled carbon nanotubes, Sol–gel technique, Volatile organohalogen compounds

## Abstract

A green, environmental friendly and sensitive method for determination of volatile organohalogen compounds was described in this paper. The method is based on a homemade sol–gel single-walled carbon nanotube/silica composite coated solid-phase microextraction to develop for sampling and analysis of Carbon tetrachloride, Benzotrichloride, Chloromethyl methyl ether and Trichloroethylene in air. Application of this method was investigated under different laboratory conditions. Predetermined concentrations of each analytes were prepared in a home-made standard chamber and the influences of experimental parameters such as temperature, humidity, extraction time, storage time, desorption temperature, desorption time and the sorbent performance were investigated. Under optimal conditions, the use of single-walled carbon nanotube/silica composite fiber showed good performance, high sensitive and fast sampling of volatile organohalogen compounds from air. For linearity test the regression correlation coefficient was more than 98% for analyte of interest and linear dynamic range for the proposed fiber and the applied Gas Chromatography-Flame Ionization Detector technique was from 1 to 100 ngmL^−1^. Method detection limits ranged between 0.09 to 0.2 ngmL^−1^ and method quantification limits were between 0.25 and 0.7 ngmL^−1^. Single-walled carbon nanotube/silica composite fiber was highly reproducible, relative standard deviations were between 4.3 to 11.7 percent.

## Background

Halogenated volatile organic compounds (HVOCs) that known as volatile organohalogen compounds or organohalogen solvents are one of the most important organic environmental and occupational pollutants, because of their vast usage and high toxicity. These compounds have at least one halogen (fluorine, chlorine, bromine, iodine) atom with vapor pressure of more than 10 Pa at 20°C [1]. They are used in workplaces and laboratories as solvents, degreasing agents, polymerization, disinfecting agents and also as clothes dry-cleaning agents. Because of high vapor pressure, they can easily be released into the environment, and have unhealthy effects on human being [2-4]. The International Agency of Research on Cancer (IARC) has classified Chloromethyl methyl ether and Trichloroethylene in group 1, Benzotrichloride in group 2A and Carbon tetrachloride in group 2B [5]. These solvents may be also, mutagenic or teratogenic as occupational pollutants [6].

There are several techniques for sampling and analysis of HVOCs. Sampling and analysis of HVOCs in the most of samples needs sample preparation and require toxic solvents consumption for extraction of methods. Time consuming procedures (for sample preparation) and low sensitivity of analysis are other disadvantages of methods. The trend for solid phase microextraction (SPME) based preconcentration methods is increasing, which do not need expensive on-line heating and enable high-yield analysis of HVOCs at trace-level concentrations. This method is one of the widely accepted and applied techniques. SPME was proposed by Pawliszyn and coworker [7] in the early 1990s. Nowadays, the SPME technique has been widely used in different fields such as environment, food, natural products, pharmaceuticals, biology, toxicology, and forensics [8]. Integrating sampling, extraction, concentration and sample introduction in a single process has been done by SPME that is mainly carry out on SPME fibers.

Up to now, several types of SPME fibers are commercially available [9,10]. Although the use of SPME fibers becomes more and more, there are some disadvantages that need to be overcome. Some drawbacks of the commercial fibers related to the shortage of proper chemical bonding of the stationary-phase coating and the relatively high thickness of the conventional fibers.

Sol–gel is a kind of technology, which is able to overpower the problems. It is a popular chemical method that offers a simple path for synthesizing new material systems and applies for surface coating. Sol–gel chemistry can efficiently incorporate inorganic compounds into organic polymeric structure in solution under mild conditions [11]. The sol–gel method is applied for the preparation of SPME fibers. Recently, many studies have been reported on the preparation of new kinds of fiber coatings for SPME and their analytical application in the pre-concentration of contaminants from environmental, biological and food samples [12,13]. Stability, polarity, thickness, surface area of the coating and the amount and rate of absorption should be considered in the design of SPME fibers. Among coating development method, the sol–gel method has drawn attention because it provides a synthetic technique for both inorganic and organic–inorganic hybrid materials. Different materials can be synthesized on the SPME fiber for increasing the sensitivity and selectivity. The sol–gel process occurs under extraordinarily mild conditions, so it produces products of various sizes, shapes and forms. Recently, Malik and coworkers established a convenient pathway to surface coatings using sol–gel technology to overcome important drawbacks of conventional SPME coatings: low recommended operating temperature, instability and swelling in organic solvents and expensive cost [14-16].

There are some advantages of the sol–gel method applying in SPME fiber coating such as: high thermal stability resulting from chemical binding of the polymeric structure; good mixing for multi-component system and possibility of creating hybrid organic–inorganic materials; and possibility to control the coating thickness.

The combination of Carbon nanotubes (CNTs) science with sol–gel chemistry essentially allows synthesizing proper sorbent [17-19] and prepared coatings to efficiently merge the advantages both from the CNTs and sol–gel technology. CNTs, essentially an allotropic form of graphitic carbon, were first described in 1991 by Iijima [20]. CNTs, which include single wall carbon nanotubes (SWCNTs) and multi-walled carbon nanotubes (MWCNTs), have captured the attention of researchers worldwide due to their unique properties. CNTs have high surface area, the ability to establish π- π interactions, excellent chemical, mechanical and thermal stability, etc., which make them very attractive as adsorbents in SPE and SPME devices for either non-polar (in the case of non-functionalized CNTs) and polar compounds for which functionalization of the tubes plays a key role in selectivity [21].

To achieve the best sampling efficiency of the SWCNTs/silica composite coated SPME, several factors affecting the sampling efficiency, such as extraction time, temperature and relative humidity inside the standard chamber, were investigated and optimized. The application of SPME and it’s newly synthesized coated fiber for the environmental and occupational assessment of some VOCs and HVOCs was the main goal of this study, so the performance characteristics of the SPME and proposed sorbent as a field sampler should be determined against atmospheric parameter such as temperature and relative humidity.

Only a few investigations have been published on the application of CNTs in the fiber coating for SPME. Despite the authors’ intensive literature review, no study combining SWCNTs with SPME as sorbent for sampling and analysis of HVOCs in air was identified. The aim of this research was to improve on previous work through a simple and practical device that can overcome remaining main problems with ordinary SPME fibers. So, we report a novel, simple and rapid method to prepare a SWCNTs SPME fiber for HVOCs samples.

## Materials and methods

### Reagents and standards

The SPME devices for manual sampling and a 75 μm commercially available CAR/PDMS coated fiber for comparison were obtained from Supelco and also prepared by modification of a commercial SPME fiber holder and assembly. SWCNTs-COOH with purity higher than 90%, with 1–2 nm O.D., 0.8–1.6 nm I.D. and length of 5–30 mm and rate of surface carbon atom 8–10 mol%, were obtained from Chengdu Organic Chemicals (Chinese Academy of Sciences). The –COOH content of SWCNTs was 2.73 wt% and special surface area(SSA) was more than 380 m^2^ g^−1^. Carbontetrachloride(CTC), Benzotrichloride, Chloromethyl methyl ether (CMME) and Trichloroethylene(TCE) with highest purity available were purchased from Sigma-Aldrich (Germany). Ultra high purity Nitrogen was obtained from Roham (Tehran, Iran). Deionized water used for preparation of SWCNTs was obtained from a TKA (Germany) ultra water system. Trifluoroaceticacid (TFA), Tetra-methylorthosilicate (TMOS) and polymethylhydrogensiloxane (PMHS) were supplied from Merck (Darmstadt, Germany). Sodium dodecyl benzene sulfunate (SDBS) was purchased from Fluka (Buchs, Switzerland).

### Instrumentation

Chromatography was performed with GC-2010 Shimadzu with a capillary column (VOCOL with 60 m × 0.25 mm × 0.25 mm) and a split-splitless injector. The column was initially set at 40°C and held at this temperature for 4 min, then ramped at 6°C min ^−1^ to 160°C and held at this temperature for 5 min, for a total runtime of 29 min. For the separation of desorbed HVOCs from the SPME, injection was performed in splitless mode at an injection port temperature of 250–290°C. The carrier gas was Nitrogen (99.999%) at a flow rate of 0.76 mL min ^−1^. A home-made chamber was used for adjustment of concentration, temperature and humidity of sample matrix. A 21- gauge needle with 12 cm length and 700 μm I.D. and a 25- gauge needle were purchased from Kosan LTD (Japan). A syringe pump, JMSSP-510 (Hiroshima, Japan), was used for providing standard concentration and determined injection of the calculated amount of HVOCs into the sampling chamber. A high volume sampling pump SKC (PA, USA) was used for drawing air through chamber.

### Fiber assembly preparation

For preparation of fiber assembly, we modified a commercial SPME fiber assembly (Supelco) as following: Placing and attaching of a fused-silica to a 25- gauge needle, inserting of them into a 21- gauge needle as a protective needle and placing of them into the black cylinder through hexagonal nut. Screwing the fiber assembly into the end of the plunger.

### SPME fiber preparation

Preparation of the sol–gel SPME fiber consists of: pretreatment of the fused-silica (FS) surface, preparation of the sol–gel solution, coating of the retreated fused-silica surface and conditioning of the coated surface. Prior to sol- gel coating, the protective polyimide layer on a 1 cm tip of a 12 cm piece of FS was removed by dipping it in acetone for several hours to expose the FS core. Then the FS was dipped in 1 M NaOH solution for 1 h, to expose the maximum number of silanol groups on the surface of the fiber, and cleaned with water then it was placed in 0.1 M HCl solution for 30 min to neutralize the excess NaOH, cleaned again, and air-dried at room temperature. The sol–gel solution was prepared as follows: 2 mg of SWCNTs with COOH group was dispersed in 50 mL of SDBS solution (5% w/v) as a surfactant in an Eppendorf vial. The obtained suspension was agitated by ultrasonic bath for 15 min and then 400 mL TMOS and 50 mL PMHS were added and the mixture was sonicated for 30 min. Afterward, 50 mL of TFA was added, and the total solution was sonicated in an ultrasonic bath for 15 min. The mixture was then centrifuged at 4000 rpm for 10 min and the top clear sol solution was removed. The activated outer surface of the FS dipped vertically into the solution, kept in it for 2 min and it was placed into GC injector at 150°C for 1 min. This coating process was repeated for several times until the desired thickness of the coating was obtained. It was experimentally approved that after several times repeating the coating process, our adsorption data were more reproducible and efficient. The sol–gel SWCNTs fiber was conditioned at 300°C under nitrogen for 1 h.

### Sampling by SPME

A home-made chamber was prepared for SPME sampling. In this chamber, a dynamic standard concentration of a predetermined amount of Carbon tetrachloride, Benzotrichloride, Chloromethyl methyl ether and Trichloroethylene were prepared with adjusted injection of each analytes using a syringe pump into a flow direction line connected to the sampling chamber. With this system, a different range of concentrations from 0.001–250 ngmL^−1^ for each analytes was achieved. The sampling temperature was at (5, 20 and 35°C) using a thermo stated plate and a visible light radiation lamp inside an additional chamber, located upstream of the sampling chamber. The temperature inside the chamber was successfully adjusted in a defined range using this temperature controller system. For adjusting relative humidity inside the chamber, a humidifier and a hygrometer system were used, and relative humidity was also successfully adjusted in two levels of 30% and 70%. For the sampling and adsorption of analytes, the fiber of the needle were inserted into the sampling chamber and the fiber was exposed to analytes for taking. A high volume sampling pump SKC (PA, USA) was used for drawing of air through chamber.

## Results and discussion

### Surface structure of the fiber

**Figure 1:**
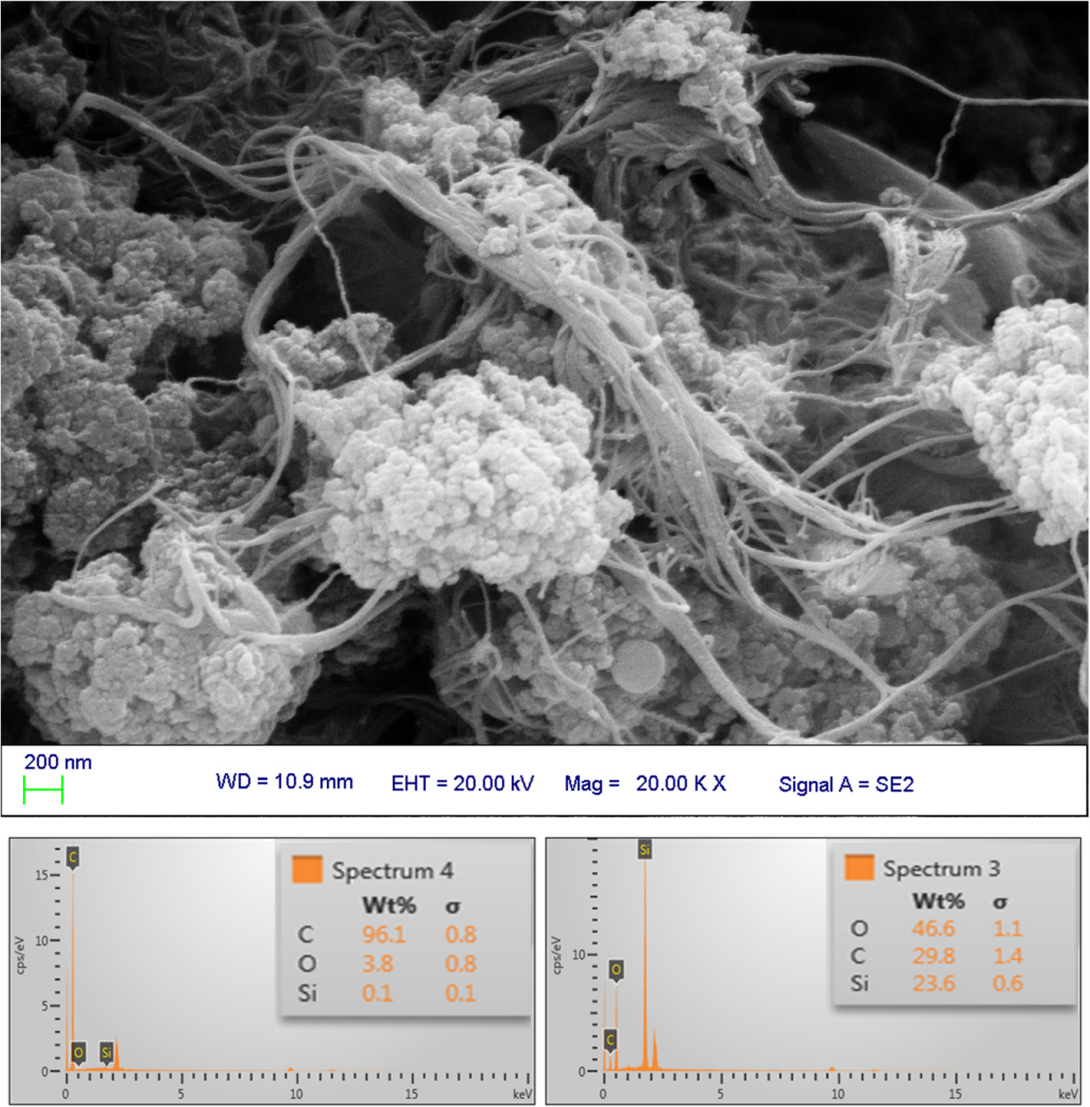
**Scanning electron micrograph and EDS analysis of the SWCNTs which used as sorbent in SPME.**

The characterization of obtained SWCNT checked by scanning electron microscopy (SEM) and the elements mass were, before and after sol–gel process were calculated by EDS analysis (Figure 1). A lot of SWCNTs/silica were observed which showed well that the SWCNTs were dissolved in the sol–gel solution and distributed within the coating.

### Extraction time

**Figure 2:**
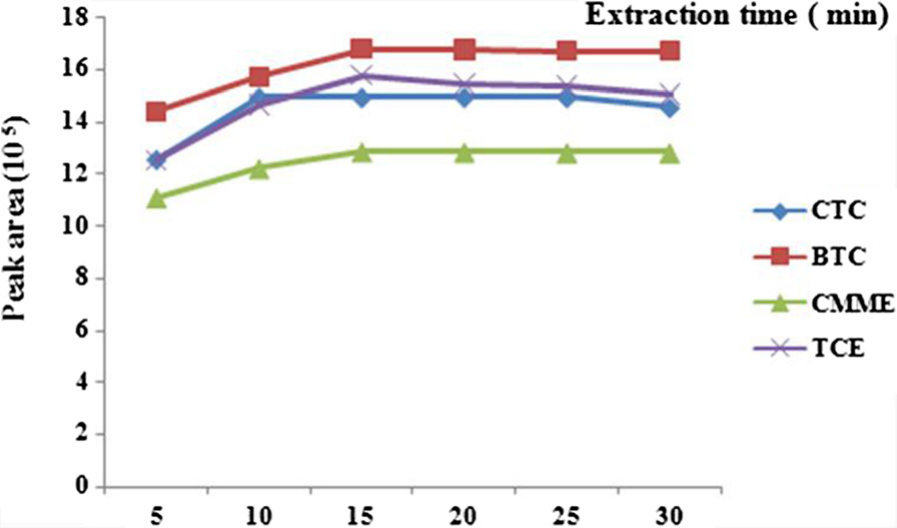
**Peak area response of SPME with SWCNTs/silica composite for extraction time.**

Mass-transfer is a time-dependent process, and its rate affects the equilibrium conditions [22]. Exposure of the fiber in gaseous sample is an important parameter in achieving distribution equilibrium of the analyte between fiber and sample; it is a decisive factor for improving the extraction efficiency [23]. Hence the extraction procedure was carried out at 5, 10, 15, 20, 25 and 30 min for determination of equilibration time and then optimum sampling time for further analysis. For this issue three consecutive sampling at each predetermined extraction time were taken and then the plot of peak area against sampling time was drawn. The results showed that with increasing sampling time the related peak area was increased until 15 min after exposing SPME fiber to the standard chamber (Figure 2). The results indicated that the SWCNTs/silica composite coated fiber had good extraction capacity and also equilibration time for the HVOCs as analytes of interest. Reaching to the equilibration time means maximum extraction capacity achieved in a short time and this could help for rapid sampling and analysis for environmental and occupational assessment of different pollutants. In this study, an extraction time of 15 min was selected as a compromise between analysis time and method sensitivity.

### Effect of relative humidity on sampling efficiency

**Figure 3:**
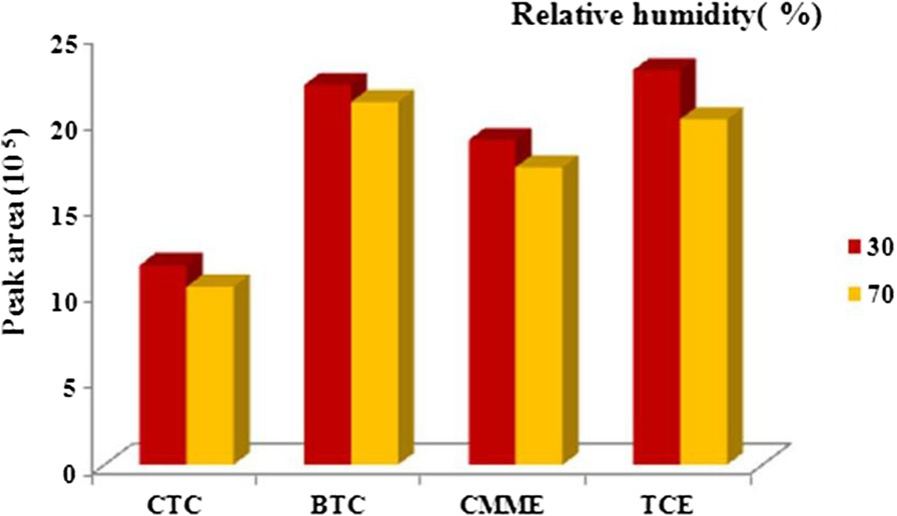
**Peak area response of SPME with SWCNTs/silica composite for effect of relative humidity on sampling efficiency.**

The possible effects of relative humidity should be assessed for using SWCNTs/silica composite coated SPME as a sampler for environmental and occupational survey. For this issue the relative humidity effects on performance of proposed sorbent was investigated at two levels of low and high humidity (30 and 70%). After adjusting the relative humidity inside the standard chamber, for each analytes of interest three consecutive analyses were performed and peak response for each levels of relative humidity were assessed (Figure 3). The results have revealed that with increase of the moisture inside the standard chamber the peak response decreased. The moisture in the air causes to stick together the sorbent particles and decreases the specific sorbent area and it may be also because of the competition between the analyte and water molecules for adsorption sites.

### Sampling temperature

**Figure 4:**
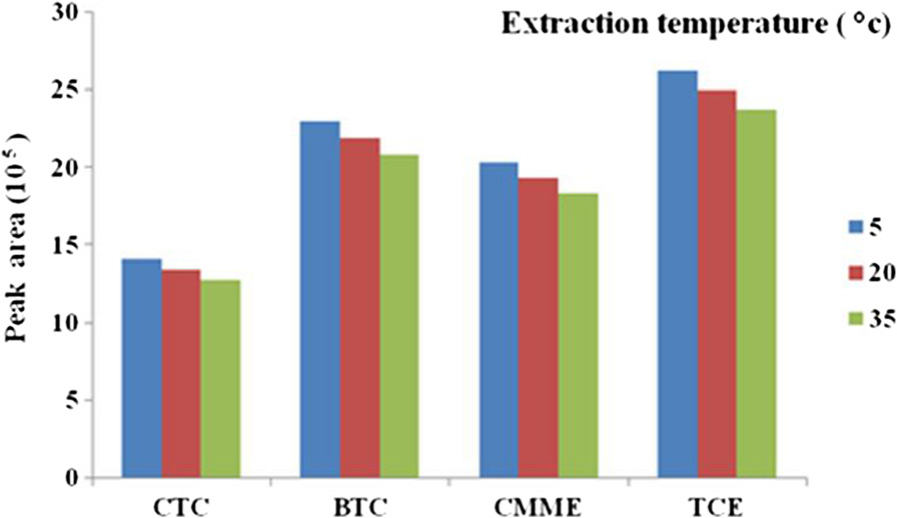
**Peak area response of SPME with SWCNTs/silica composite for effect of temperature on sampling efficiency.**

The temperature is a fundamental parameter governing the efficiency of the process. Utilizing the temperature influence is rather restricted, as its increase leads to improvement of the rates of mass transport between the phases, at the same time worsening the partition coefficient(s) [24]. According to SPME theory, the fiber equilibration process is an exothermic process and increasing the sample temperature will decrease both analyte recovery and extraction time [25]. For using SPME with proposed sorbent as field sampler it is necessary for investigation of atmospheric temperature on performance characteristics and sampling efficiency. The effects of extraction temperature on the extraction recoveries of CTC, TCE, BTC and CMME from standard chamber with SPME coated with proposed sorbent were investigated. In order to obtain the extraction temperature profile, the analytical procedure was performed over an extraction time of 15 min. Extraction temperatures of 5, 20 and 35°C were investigated. These amounts as sampling temperature are close to the environmental and occupational atmospheric condition for using SPME with proposed sorbent as field sampler (Figure 4). The results showed that with increasing temperature, the peak response will be decreased accordingly and temperature has effect on sampling efficiency of SPME coated with SWCNTs/silica composite as fiber. The effect of temperature on the adsorption mechanism of an analyte onto any adsorbent media is well-known and understood. This reality proves that in an adsorption mechanism, temperature has an adverse effect on the extraction efficiency. In fact increasing of temperature causes increasing of vapor pressure and following increasing of volatility of compounds.

### SPME storage time

**Figure 5:**
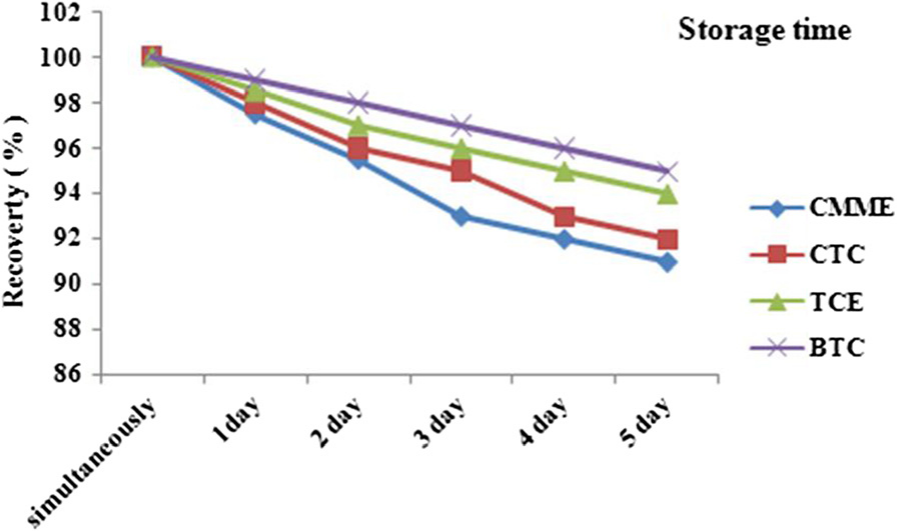
**Peak area response for analytical performance of SPME with SWCNTs/silica composite at different levels of storage time.**

When using SPME as field sampler, shipping may happened and the time between sampling and analysis should be considered and sustainability of analytes adsorbed by the sorbent must be evaluated. For this issue at laboratory temperature (25°C) and at the optimum condition temperature and relative humidity, sampling was carried out and analysis was performed at the time between simultaneously to 5 days after sampling. The results for storage time capabilities of the SPME coated with proposed sorbent compared to the simultaneous analysis for the determination of the losses analytes (Figure 5). The results for storage analysis demonstrated that the SWCNTs/silica composite has a good storage capability due to the strong affinity to the HVOCs.

### Carryover of SWCNTs/silica composite coated SPME

Before considering any results of the parameters related to GC response, the carryover of the analytes of interest on SPME coated with SWCNTs/silica composite should be determined. Investigation of the carryover percentage is necessary for determining conditioning time and prevention of memory effect on further analysis. For investigation of carryover, after desorption in time allocated, SPME with proposed sorbent inserted into GC injection port for additional desorption and determining amounts of analytes remains on sorbent surface. For this issue SPME inserted to the GC injection port for 1 to 4 min. Results revealed that after 2 min as conditioning time, the sorbent was completely free of analytes. So this time was selected as conditioning time of SPME coated fiber for prevention of memory effect on further analysis.

### Desorption time and temperature

**Figure 6:**
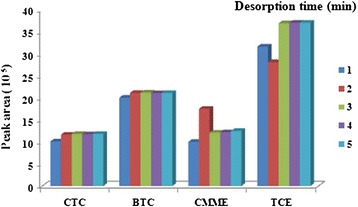
**Peak area response for analytical performance of SPME with SWCNTs/silica composite at different levels of desorption time.**

Desorption time and temperature are two important analytical performances that deal to the time of analysis and life span of the sorbent. Increasing desorption time can reduce the carryover and memory effect of the adsorbed analytes on sorbent surface but also can reduce the life span of the sorbent and the time that sorbent could be applied for consecutive analysis. In order to examine this performance parameter, sampling was performed at optimum condition and then analysis was carried out at the time between 1 to 5 min inside GC injection port. The peak responses acquired at each desorption time, were plotted. The minimum desorption time that accompany with maximum peak response was selected as optimum desorption time which reduces the analysis time and increases the life time of the sorbent as well. The (Figure 6) shows the desorption time investigation result.

**Figure 7:**
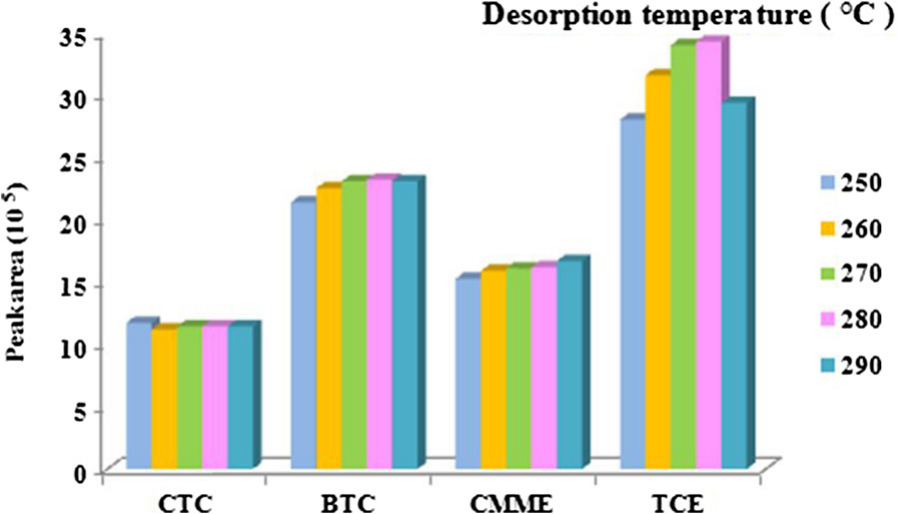
**Peak area response for analytical performance of SPME with SWCNTs/silica composite at different levels of desorption temperature.**

Much like desorption time, desorption temperature also investigated as analytical performances in this study. Increasing desorption temperature can cause reduction desorption time with minimum carryover but also can reduce the life time of the sorbent. All sorbents using as sampling media has limited thermal stability and extensive temperature in heating zone of GC injector can destroy the sorbent structure gradually. For investigation desorption temperature after sampling by SPME at optimum sampling condition inside standard chamber from analytes of interest at constant concentration of 100 ngmL^−1^, analysis was performed at the GC injection port temperature of 250–290°C and peak responses were plotted for each temperature and minimum temperature with maximum peak response was selected as optimum desorption time for further analysis. The results in (Figure 7) revealed efficient temperatures for thermal desorption of SPME coated with SWCNTs/silica composite as coating fiber is 280°C and increasing temperature doesn’t have any effect on peak responses.

### Analytical performances

#### Limit of detection and limit of quantitation

The limits of detection (LODs), defined as the concentration of analytes in samples which cause a peak with a signal-to-noise ratio of three, were also determined. In order to calculate them, with adjusting the syringe pump injection of interest analytes into standard chamber on lowest possible amount and also with dilution of analytes in suitable solvents with less interfere with analytes and GC performance, concentrations of analytes in ppb level acquired. After sampling by SPME coated with proposed sorbent the samples introduced to GC injection port and dilution of analytes in standard chamber as well as introducing the sample to GC continued until for each analytes of interest the ratio for signal to noise in chromatogram reach to three. Then the corresponding concentration to obtained peak area reported as limit of detection for SPME coated with each sorbents. Limit of quantitation (LOQ) also calculated as concentration relevant to peak area of each analytes of interest with ratio of signal to noise of ten. According to the ICH (International Conference on Harmonization of Technical Requirements for Analytical Methods) guideline for analytical method validation, limit of quantification (LOQ) for each analyte was determined as the lowest concentration on the calibration curve with a precision of less than 20% coefficient of variation (CV%) and an accuracy of 80–120% [26]. Same as LOD for LOQ, with adjusting injection rate of syringe pump also dilution of analytes and continuous sampling and analysis of each analytes was used.

#### Linearity and repeatability

In order to evaluate the practical applicability of the SPME coated with the proposed sorbent, performance parameters such as linearity and repeatability were measured under optimum extraction conditions using 8 points at high, medium, and low concentrations levels in the range of 0.1–100 ngmL^−1^ (0.1, 0.5, 1, 5, 10, 25, 50, and 100 ngmL^−1^) with triple consecutive measurements for each analytes were applied. Linear dynamic ranges (LDRs) of calibration curves for coated with the proposed sorbent, were determined for each analyte of interest. For comparison of the results, commercial CAR/PDMS fiber was selected. Some other studies also reported that CAR/PDMS has high affinity toward halogenated compounds [27]. As shown in Table 1, the calibration curves illustrated good linearity with appropriate values of the correlation coefficient (r^2^ > 0.98).

**Table 1:** **Some analytical data obtained by using the sol–gel SWCNT/SILICA composite fiber and GC for four Organohalogen compounds**

**Compound**	**Fiber type**	**Range (ngmL** ^**−1**^ **)**	**LDR (ngmL** ^**−1**^ **)**	**r** ^**2**^	**RSD (%)**	**LOD (ngmL** ^**−1**^ **)**	**LOQ (ngmL** ^**−1**^ **)**
TCE	SPME-SWCNTs/Silica composite	0.1-100	1-90	0.9864	5.6	0.18	0.5
	SPME-CAR/PDMS	0.1-100	0.1-100	0.9832	7.5	0.21	0.5
BTC	SPME-SWCNTs/Silica composite	0.1-100	1-80	0.9841	9.5	0.2	0.7
	SPME-CAR/PDMS	0.1-100	0.1-100	0.9851	6.5	0.23	0.9
CTC	SPME-SWCNTs/Silica composite	0.1-100	1-70	0.9815	4.3	0.09	0.25
	SPME-CAR/PDMS	0.1-100	0.1-100	0.9832	4	0.12	0.38
CMME	SPME-SWCNTs/Silica composite	0.1-100	1-100	0.9862	11.7	0.15	0.48
	SPME-CAR/PDMS	0.1-100	0.1-100	0.9871	9.2	0.18	0.52

Repeatability was also determined by calculating the relative standard deviation (RSD) of peak responses of inters-SPME coated with SWCNTs/silica composite sampling for analytes of interest at five concentration levels of 1, 10, 50, 100, and 250 ngmL^−1^ (n = 5) under optimized extraction conditions. The results for the relative standard deviation also demonstrate a reasonable repeatability for the proposed SPME method.

## Conclusion

The results showed that SPME coated with newly synthesized sorbent of SWCNTs/silica composite with sol–gel technique offered an attractive alternative to commercially available fibers for the analysis of HVOCs in environmental and occupational samples. The fiber exhibited relatively good repeatability and high thermal stability. Sampling temperature and humidity, storage time and GC operation parameters as analytical performances, were investigated, and sorbent performance were evaluated. The proposed fiber has several advantages in simplicity in sample preparation, preconcentration and analysis of HVOCs.

This method is rapid, simple, inexpensive, providing a high degree of sensitivity and pre-concentration. The operation is easy to handle because the sampling and analysis will performed in single step. Under the optimized conditions, this technique provided limits of quantitation in the 0.25-0.7 ngmL^−1^ range and acceptable precision and linearity. CNTs coated SPME fiber were excellent coating materials for their strong physical adsorption ability to various analytes, high extraction efficiencies for both polar and non-polar compounds, good thermal stability to resist 350°C [28-30]. Combining CNTs advantages with sol–gel techniques and producing silanated CNTs sorbent is a novelty of this work and can be used as sorbent in SPME. Coupling SPME coated with single walled carbon nanotubes/silica composite as coating fiber with GC-FID provided a powerful technique for sampling and analysis of occupational/environmental pollutants in air.
